# Complete chloroplast genome of the endemic species *Hoya lockii* (Apocynaceae) from Viet Nam

**DOI:** 10.1080/23802359.2026.2658954

**Published:** 2026-04-17

**Authors:** Nga Thi Thu Nguyen, Changyoung Lee, Manivanh Yongsa, Quan Huu Nguyen, Sangjin Jo, Thuong Danh Sy, Mau Hoang Chu

**Affiliations:** a Faculty of Biology, Thainguyen University of Education, Thainguyen City, Viet Nam; b International Biological Material Research Center (IBMRC), Korea Research Institute of Bioscience and Biotechnology (KRIBB), Daejeon, Republic of Korea

**Keywords:** Chloroplast genome, endemic species, *Hoya lockii*, phylogeny, Viet Nam

## Abstract

Here we report the first chloroplast genome of Hoya lockii V.T. Pham & Aver., sequenced as a circular DNA molecule (177,063 bp) comprising a large single-copy region (91,635 bp), a small single-copy region (2,242 bp), and two inverted repeats (41,593 bp), with a GC content of 36.9%. A total of 144 genes were annotated, including 98 protein-coding, 38 tRNA, and eight rRNA genes. Phylogenetic reconstruction based on 79 protein-coding genes from 50 Apocynaceae species placed H. lockii as sister to Hoya exilis with strong bootstrap support (93%). These results indicate pronounced SSC contraction and IR expansion, consistent with Hoya evolution.

## 1. Introduction

Chloroplasts are essential for photosynthesis and metabolic processes in plants (Daniell et al. 2016; Yun et al. 2021). Chloroplast (cp) genomes are widely used for phylogenetic reconstruction, evolutionary studies, and species identification (Sugiura 1995; Finkeldey and Gailing 2013; Leister 2013). In *Hoya*, plastome-based analyses have proven valuable for resolving evolutionary relationships, and structural variations such as inverted repeat (IR) expansion and contraction provide necessary phylogenetic signals (Zheng et al. 2022). Recent studies on *Hoya* plastomes have further shown that IR boundary dynamics contribute significantly to plastome size variation and phylogenetic inference within this genus (Zhu et al. 2016; Wei et al. 2020; Odago et al. 2021).

Within the genus *Hoy*a (Apocynaceae), previous research has revealed plastomes ranging from 175,404 to 179,069 bp, characterized by inverted repeat (IR) expansion and small single-copy (SSC) contraction (Tan et al. 2018; Wei et al. 2020; Odago et al. 2021; Rodda and Niissalo 2021). However, despite recent progress, plastome data for *Hoya* remain limited, as complete chloroplast genomes have so far been reported for only a small number of species (more than 30 species of *Hoya*) (Odago et al. 2021). Meanwhile, the genus *Hoya* comprises 562 accepted species, according to Plants of the World Online (2025) (https://powo.science.kew.org/taxon/urn:lsid:ipni.org:names:60437256-2). The currently available restricted taxon sampling limits comprehensive assessments of plastome evolution and hampers robust phylogenetic inference across *Hoya.*

*Hoya lockii* V.T. Pham & Aver. 2012, described from Viet Nam in 2012 (The and Averyanov 2012), is a rare endemic species classified as Critically Endangered on the IUCN Red List. Despite its conservation importance, no plastome data have been reported. The present study aimed to assemble and annotate the complete cp genome of *H. lockii*, identify divergence hotspots, and determine its phylogenetic placement within Apocynaceae. The results provide valuable genomic information for taxonomy, molecular barcoding, and conservation genetics.

## 2. Materials and methods

**Figure 1.**
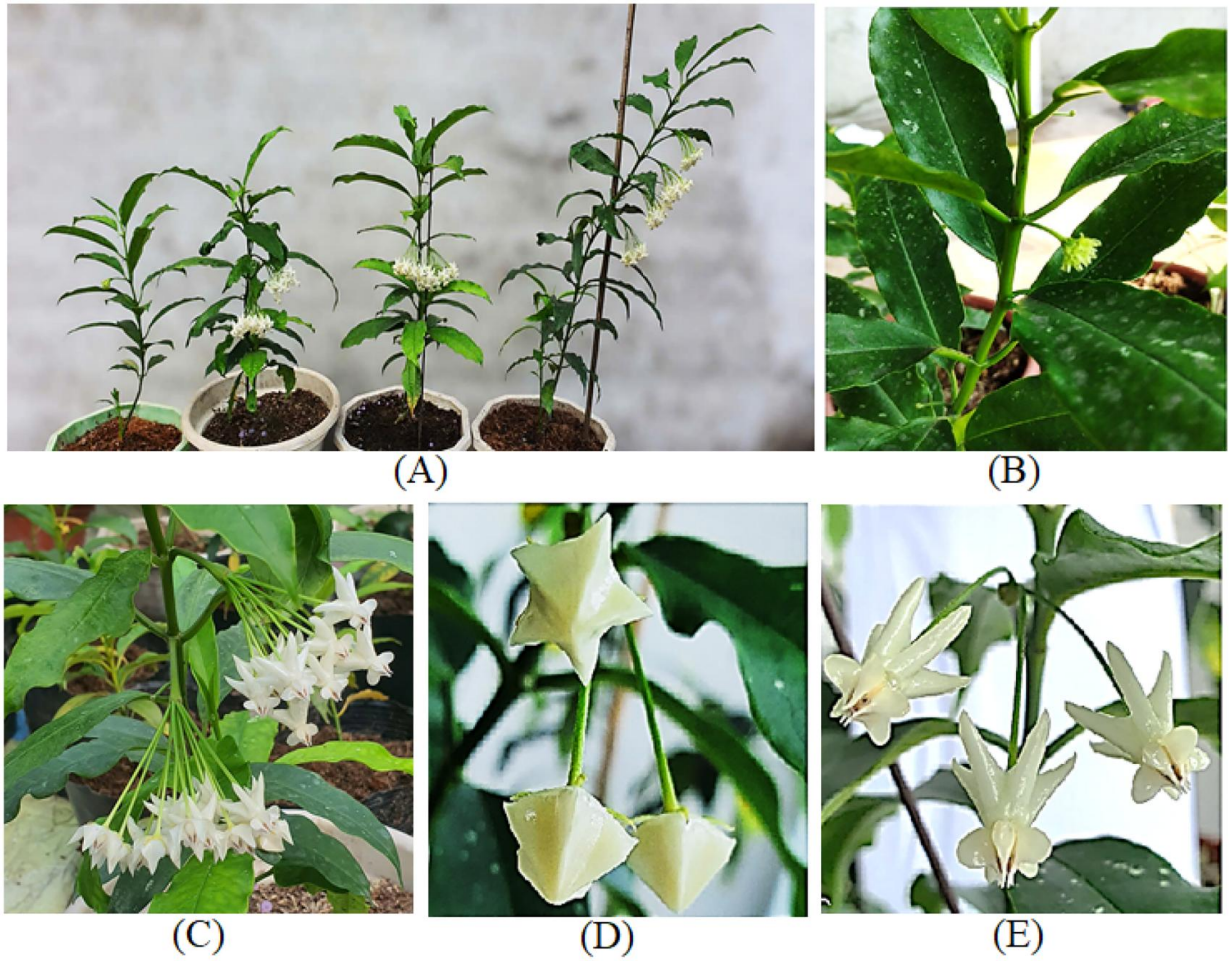
*Hoya lockii* were taken by Nguyen, T.T.N. at the Faculty of Biology experimental garden at Thai Nguyen University of Education, Viet Nam (21° 35′ 55.0921″ N, 105° 49′ 25.5569″). *H. lockii* 40–80 cm tall. Stems are smooth, dark green when young, turning pale gray with age. Leaves opposite, ovate to elliptic with an acuminate, caudate apex and entire margin. Inflorescences umbellate, bearing 13–21 flowers; corolla thick, dull white, glossy, and smooth. Ovary superior, bicarpellate. Fine hairs cover most plant parts except the leaf blades and corolla. (A) Plants propagated *in vitro* and cultivated in a greenhouse; (B) Branch with leaves and developing inflorescence; (C) Branch with leaves and mature flower clusters; (D) Inflorescence bearing a flower bud; (E) Fully developed flowers.

A specimen of *H. lockii* was collected from Dong Son, Ky Thuong Nature Reserve, Quang Ninh Province, Viet Nam (21.1189° N, 107.0631° E), and Assoc. Prof. Sy T.D., Department of Botany, authenticated the taxonomic identification. A specimen was deposited at the Faculty of Biology, Thai Nguyen University of Education, Viet Nam (Nguyen, T.T.N.; ngantt.bio@tnue.edu.vn) under voucher number HL2022-VN01 (Figure 1), and the dataset of *Hoya lockii* can be found at https://data.mendeley.com/datasets/7gbt9kr5r3/1.

Total genomic DNA was extracted using the DNeasy Plant Mini Kit (Qiagen, Germany). High-quality DNA was sequenced on the Illumina NovaSeq 6000 platform (150 bp paired-end reads). De novo assembly was conducted using NOVOPlasty v4.3.1 (Dierckxsens et al. 2017), with *Hoya angustifolia* (GenBank OL754669.1) as a reference. Annotation was performed with Geneious Prime v2023.2.1 (Kearse et al. 2012) and verified using tRNAscan-SE 2.0 (Chan et al. 2021). Raw reads were quality-filtered using BBDuk and BBNorm, then assembled using NOVOPlasty and Geneious Prime. Resulting contigs were compared to generate the final plastome. Annotation was performed in Geneious, with tRNAs verified by tRNAscan-SE. The genome map was visualized in OGDRAW v1.3.1 (Greiner et al. 2019). Simple sequence repeats (SSRs) were identified using Phobos v3.3.12 (Leese et al. 2008; Mayer et al. 2010), and large sequence repeats were detected with REPuter (Kurtz et al. 2001). Comparative genome alignments were performed in Mauve, while IR boundary visualization used Irscope (Amiryousefi et al. 2018). Nucleotide diversity was calculated with DnaSP v6.12.03 (Rozas et al. 2017).

Phylogenetic reconstruction was based on 79 concatenated protein-coding genes from 50 Apocynaceae species using MAFFT (Katoh and Standley 2013) and RaxML (Stamatakis 2014) under the GTR+I + G model with 1,000 bootstrap replicates. The GTR+I + G model, commonly used for large concatenated plastome datasets, was applied as a general time-reversible model accounting for among-site rate heterogeneity. Outgroups included *Cynanchum sibiricum, C. wilfordii*, and *Vincetoxicum shaanxiense*.

## 3. Results

**Figure 2.**
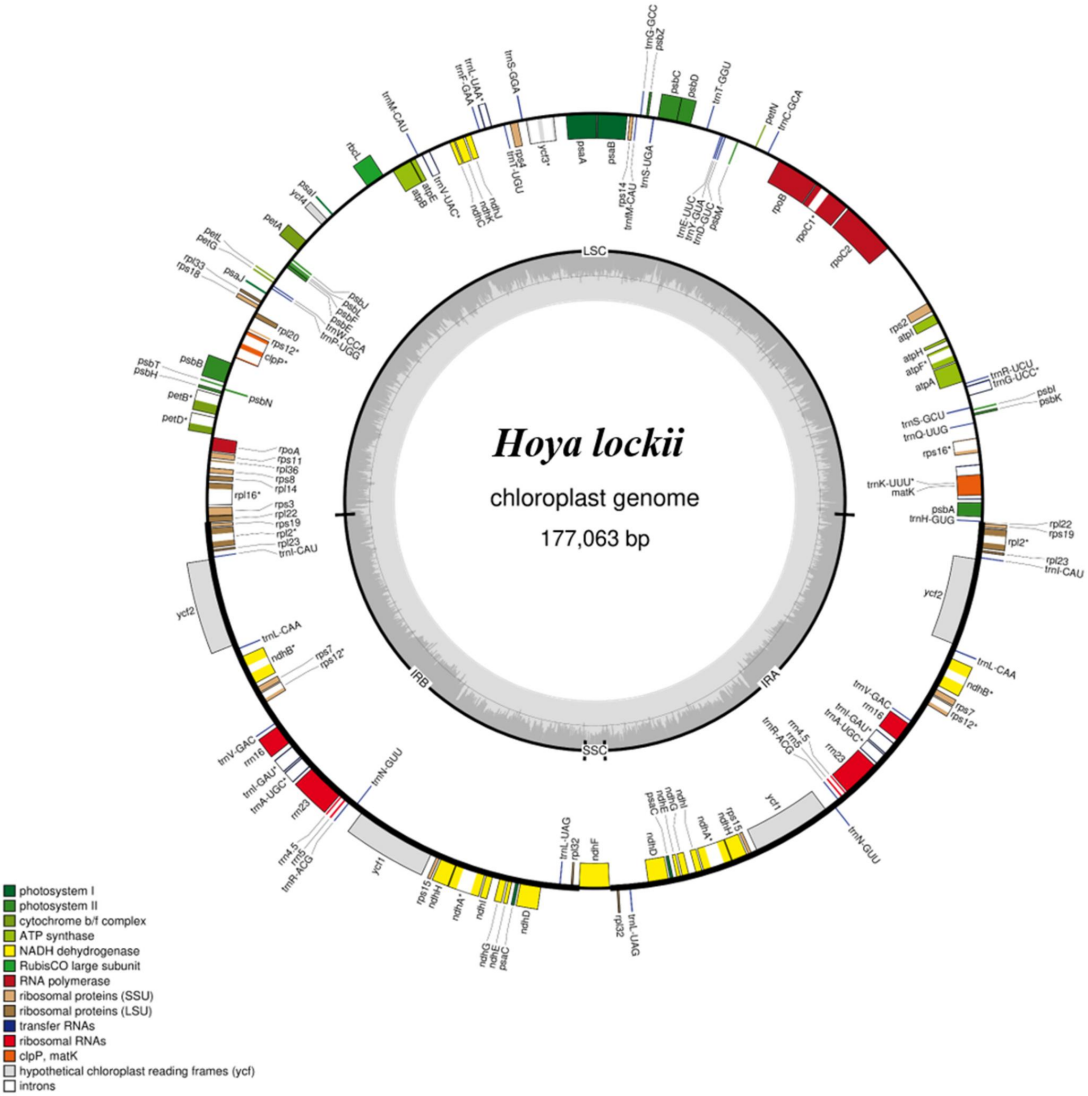
Circular map of the cp genome of *H. lockii* from Viet Nam. The inner gray circle represents the AT content, while the darker gray circle indicates the GC content. Genes transcribed clockwise are shown inside the circle, whereas those transcribed counterclockwise are displayed on the outside. Genes belonging to different functional groups are color-coded. The inner concentric rings illustrate the typical quadripartite structure of the cp genome, comprising the LSC, SSC, and IR regions.

The complete chloroplast genome of *H. lockii* is 177,063 bp long, containing a large single-copy (91,635 bp), a small single-copy (2,242 bp), and two inverted repeats (41,593 bp), with 36.9% GC content (Figure 2). In terms of sequencing coverage, the assembled genome shows a minimum read depth of approximately 950×, a maximum of 3,120×, and an average of about 2,200× (Figure S1), indicating high-quality and evenly distributed sequencing data across the genome. A total of 144 genes were annotated: 98 protein-coding genes (PCGs), 38 tRNAs, and eight rRNAs. Eighteen genes contained introns, and 27 were duplicated in IR regions (Tables S1 and S2). Additionally, the *H. lockii* chloroplast genome includes 11 *cis*-splicing genes, *rps16, atpF, rpoC1, ycf3, clpP, petB, petD, rpl16, rpl2, ndhB,* and *ndhA* (Figure S2) and one trans-splicing gene, *rps12* (Figure S3). The genome sequence was deposited in GenBank under accession OR475243.1.

A total of 94 SSRs and 181 large repeats were identified, mainly in the LSC region. A total of 94 SSRs were identified in the chloroplast genome of *H. lockii,* dominated by mononucleotide A/T-rich repeats. Dinucleotide repeats were less common, while tri-, tetra, and pentanucleotide motifs occurred at low frequencies with limited copy numbers (Figure S4A). Region-specific analysis revealed that SSRs were most abundant in the LSC region, which contained 71 SSRs. The IR region contained 23 SSRs. In contrast, no SSRs were detected in the SSC region (Figure S4B). Functional categorization showed that SSRs were predominantly located in intergenic spacer (IGS) regions (65 SSRs), followed by intronic regions (17 SSRs), and protein-coding gene (PCG) regions with 12 SSRs (Figure S4C). Large sequence repeats (LSRs) were also analyzed, revealing a total of 181 repeats, including 39 palindromic, 100 forward, and 42 reverse repeats. No complementary repeats were identified (Figure S5). The sizes of these repeat units ranged from 20 bp to 134 bp. The IR regions of the *H. lockii* plastome exhibited conserved boundaries comparable to those reported in other *Hoya* species, with no notable expansion or contraction events. The SSC region contains only one *ndhF* gene (Figure S6).

**Figure 3.**
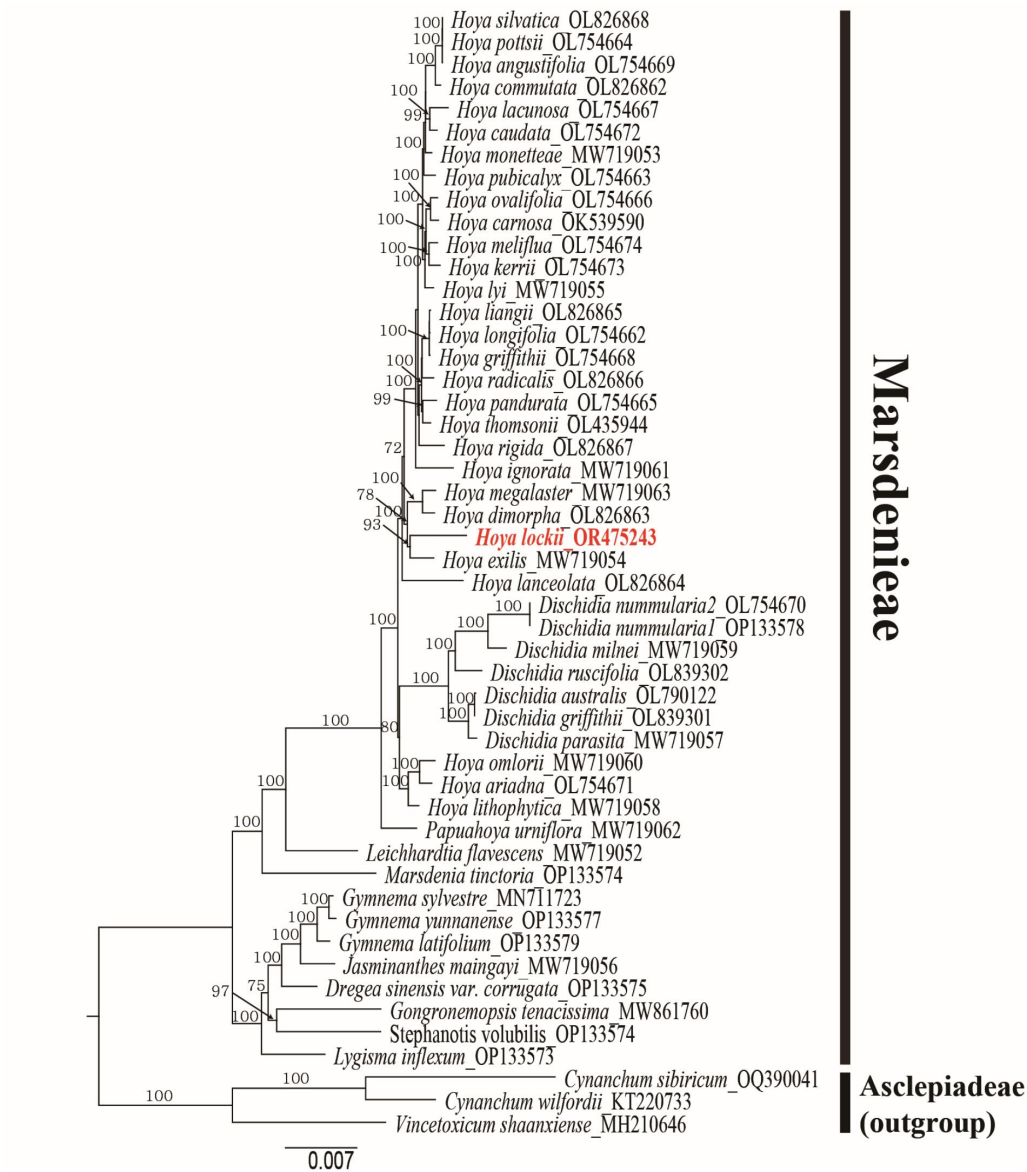
Maximum likelihood (ML) phylogenetic tree of 50 Apocynaceae species inferred from 79 concatenated protein-coding genes (total alignment length: 75,302 bp) of the chloroplast genomes. The tree was reconstructed using RAxML under the GTR+G model with 1,000 bootstrap replicates, with a final log-likelihood value of −199041.644743. Bootstrap support values (%) are shown above the corresponding nodes. The following sequences with GenBank accession were used: *Hoya lockii* V.T. Pham & Aver. OR475243.1; *H. angustifolia* Elmer. OL754669.1, *H. ariadna* Decne. L754671.1, *H. caudata* Hook.f. OL754672.1, *H. commutata* M.G.Gilbert & P.T.Li, OL826862.1, *H, dimorpha* F.M.Bailey OL826863.1, *H. kerrii* Craib OL754673.1, *H. lacunosa* Blume OL754667.1, *H. lanceolata* Wall. ex D.Don OL826864.1, *H. liangii* Tsiang OL826865.1, *H. longifolia* Wall. ex Wight OL754662.1, *H. meliflua* (Blanco) Merr. OL754674.1, *H. ovalifolia* Wight & Arn. OL754666.1, *H. pandurata* Tsiang OL754665.1, *H. pottsii* Lindl. OL754664.1, *H. pubicalyx* Merr. OL754663.1, *H. radicalis* Y.Tsiang & P.T.Li OL826866.1, *H. rigida* Kerr OL826867.1, *H. silvatica* Y.Tsiang & P.T.Li OL826868.1, *H. thomsonii* Hook.f. OL435944.1, *D. australis* Costantin OL790122.1, *D. griffithii* Hook.f. OL839301.1, *D. nummularia* R.Br. OL754670.1, *D. ruscifolia* Decne. ex Becc. OL839302.1 (Odago et al. 2021); *H. exilis* Schltr. MW719054.1, *H. ignorata* T.B.Tran, Rodda, Simonsson & Joongku Lee, MW719061.1, *H. lithophytica* Kidyoo MW719058.1, *H. lyi* H.Lév. MW719055.1, *H. megalaster* Warb. ex K.Schum. & Lauterb. MW719063.1, *H. monetteae* T.Green, MW719053.1, *H. omlorii* (Livsh. & Meve) L.Wanntorp & Meve MW719060.1, *D. milnei* Hemsl. MW719059.1, *D. parasita* (Blanco) Arshed, Agoo & Rodda MW719057.1, *J. maingayi* (Hook.f.) Rodda MW719056.1, *L. flavescens* (A.Cunn.) P.I.Forst. MW719052.1, *Papuahoya urniflora* (P.I.Forst.) Rodda & Simonsson MW719062.1 (Rodda and Niissalo 2021); *H. carnosa* (L.f.) R.Br. OK539590.1; *D. nummularia* R.Br. OP133578.1, *S. volubilis* (L.f.) S. Reuss, Liede & Meve OP133576.1, *G. yunnanense* Tsiang OP133577.1, *G. latifolium* Wall. ex Wight OP133579.1, *D. sinensis var. corrugata* (C.K. Schneid.) Y. Tsiang & P.T.Li OP133575.1, *L. inflexum* (Costantin) Kerr OP133573.1; *V. shaanxiense* Meve & Liede MH210646.1 (Rao et al. 2020); *G. sylvestre* (Retz.) R.Br. ex Sm. MN711723.1; *Gongronemopsis tenacissima* (Roxb.) S.Reuss, Liede & Meve MW861760.1; *C. wilfordii* (Maxim.) Hook.f. KT220733.1 (Jang et al. 2016); *C. sibiricum* Willd. OQ390041.2. The chloroplast genome of *Hoya lockii* generated in this study is highlighted in red. *C. sibiricum, C. wilfordii*, and *V. shaanxiense* (tribe Asclepiadeae) were used as outgroups. The scale bar indicates the evolutionary distance measured as the number of nucleotide substitutions per site. In this tree, a scale bar value of 0.007 corresponds to 0.007 substitutions per site, or approximately 0.7% sequence divergence. Therefore, a branch length equal to the scale bar represents about 0.7% nucleotide difference between sequences.

Codon usage frequencies of 98 PCGs were examined by calculating the relative synonymous codon usage (RSCU) values. The PCGs comprised a total of 30,585 codons (Table S3). Sliding-window analysis showed high nucleotide diversity in *trnS–trnG, rpl16 intron, psbZ–rps14, rbcL–accD*, and *ndhF*, suggesting these loci as hypervariable hotspots and potential barcodes (Figure S6). Phylogenetic analysis using 79 concatenated PCGs from 50 Apocynaceae plastomes supported *H. lockii* as a sister species to *H. exilis* with 93% bootstrap support, consistent with previous phylogenetic frameworks. The monophyly of the tribe Marsdenieae and the close relationship between *Hoya* and *Dischidia* were also confirmed (Figure 3).

## 4. Discussion and conclusion

The plastome of *H. lockii* exhibits a typical quadripartite structure with pronounced contraction of the SSC region and expansion of the IRs, consistent with previous reports in *Hoya* (Odago et al. 2021; Rodda and Niissalo 2021). However, the extent of these features is species-specific, with an extremely reduced SSC (∼2.2 kb) and markedly expanded IRs (∼41 kb each), representing one of the most pronounced configurations reported in the genus. Detailed analysis of IR/SSC junctions revealed lineage-specific boundary shifts, particularly involving *ycf1* and *ndhF*, which are partially or fully incorporated into the IR regions. This pattern distinguishes *H. lockii* from closely related species such as *H. exilis* and suggests ongoing plastome structural evolution within the genus.

Comparative analyses identified several variable intergenic spacers (*trnS–trnG*, *rps16–trnQ*, *rbcL–accD*, and *ndhF*) exhibiting moderate to high sequence divergence, highlighting their potential as candidate markers for species identification and phylogenetic studies. Phylogenetic reconstruction based on 79 plastid protein-coding genes provided strong support for a sister relationship between *H. lockii* and *H. exilis*. However, plastome datasets for *Hoya* remain limited, and therefore, the phylogenetic results should be considered preliminary. Broader taxon sampling will be essential to improve resolution and clarify deeper relationships within the genus.

In conclusion, this study reports the first complete plastome of *H. lockii* and highlights species-level variation in IR/SSC architecture, providing valuable genomic resources for future research in taxonomy, plastome evolution, and conservation genetics.

## Supplementary Material

Supplementary Nguyen TTN_R.docx

## Data Availability

The genome sequence data that support the findings of this study are openly available in GenBank of NCBI at https://www.ncbi.nlm.nih.gov/ under accession no. OR475243.1. The associated BioProject, Bio-Sample, and SRA numbers are PRJNA1348954, SAMN52922617, and SRR35878530, respectively.
