# Supplementary material for: Complete chloroplast genome of the endemic species Hoya lockii (Apocynaceae) from Viet Nam
Source: Mitochondrial DNA B Resour. 2026 Apr 17;11(5):653–8. doi: 10.1080/23802359.2026.2658954 (PMC13094257; doi:10.1080/23802359.2026.2658954)
Supplement: Supplementary Nguyen TTN_R.docx [file TMDN_A_2658954_SM8003.docx]

**Supplementary**

**
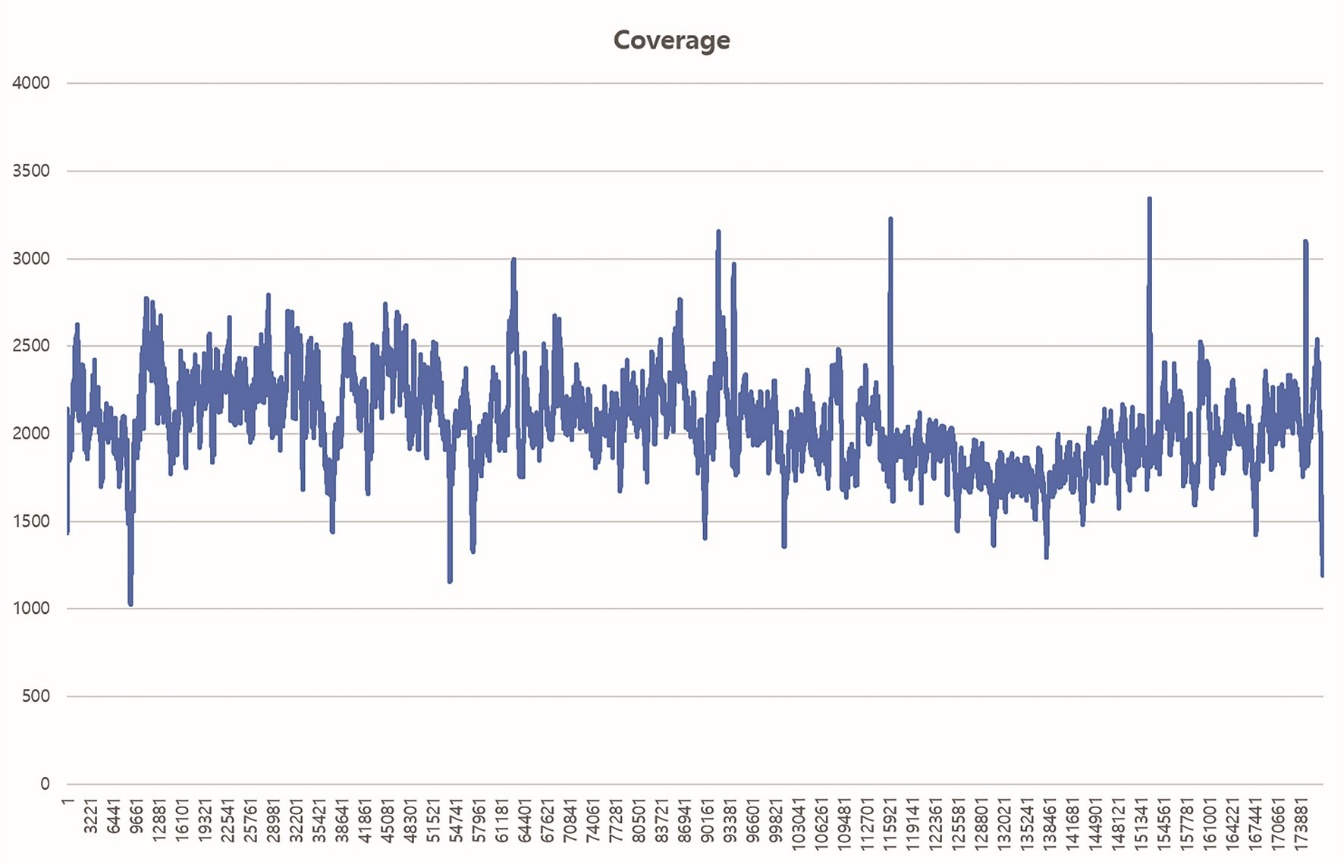
**

**Figure S1.** **Sequencing coverage across the complete chloroplast genome of *Hoya lockii* V. T. Pham & Aver.** The figure illustrates the sequencing depth along the chloroplast genome of *Hoya lockii*, showing high and relatively uniform coverage (>1000×) across the large single-copy (LSC), small single-copy (SSC), and inverted repeat (IR) regions, ensuring the accuracy and completeness of the genome assembly.

**Table S1.** Summary of the chloroplast genome of *H. lockii* species

| **Genome features** | ***H. lockii*** |
| --- | --- |
| Genome size (bp) | 177,063 |
| LSC size (bp) | 91,635 |
| SSC size (bp) | 2,242 |
| IR size (bp) | 41,593 |
| GC content (%) | 36.9 |
| No. of genes | 144 |
| No. of protein-coding genes | 98 |
| No. of tRNA genes | 38 |
| No. of rRNA genes | 8 |

**Table S2.** Genes in the *H. lockii* chloroplast genome

| **Category of genes** | **Group of genes** | **Name of genes** |
| --- | --- | --- |
| Photosynthesis | Subunits of ATP synthase | *atpA, atpB, atpE, atpF*^a^*, atpH, atpI* |
|  | Subunits of NADH-dehydrogenase | *ndhA*^a^ *(x2), ndhB (x2)*^a^*, ndhC, ndhD (x2), ndhE (x2), ndhF, ndhG (x2), ndhH (x2), ndhI (x2), ndhJ, ndhK* |
|  | Subunits of cytochrome b/f complex | *petA, petB*^a^*, petD*^a^*, petG, petL, petN* |
|  | Subunits of photosystem I | *psaA, psaB, psaC (x2), psaI, psaJ* |
|  | Subunits of photosystem II | *psbA, psbB, psbC, psbD, psbE, psbF, psbH, psbJ, psbK, psbL, psbM, psbN, psbT, psbZ* |
|  | Subunit of rubisco | *rbcL* |
| Transcription and translation | Large subunit of ribosome | *rpl14, rpl16*^a^*, rpl2 ^a^ (x2), rpl20, rpl22, rpl23 (x2), rpl32 (x2), rpl33, rpl36* |
|  | DNA dependent RNA polymerase | *rpoB, rpoA, rpoC1*^a^*, rpoC2* |
|  | Small subunit of ribosomal proteins | *rps11, rps12, rps14, rps15, rps16*^a^*, rps18, rps19* (x2)*, rps2, rps3, rps4, rps7* (x2)*, rps8* |
|  | rRNA Genes | *rrn23* (x2)*, rrn16* (x2)*, rrn5* (x2)*, rrn4.5* (x2) |
|  | tRNA Genes | *trnA-UGC ^a^ (x2), trnC-ACA^a^, trnC-GCA, trnD-GUC, trnE-UUC ^a^ (x2), trnF-GAA, trnG-GCC, trnH-GUG, trnK-UUU^a^, trnL-CAA (x2), trnL-UAA^a^, trnL-UAG (x2), trnM-CAU (x2), trnN-GUU (x2), trnP-UGG, trnQ-UUG, trnR-ACG (x2), trnR-UCU, trnS-CGA^a^, trnS-GCU, trnS-GGA, trnS-UGA, trnT-GGU, trnT-UGU, trnV-GAC (x2), trnW-CCA, trnY-GUA* |
|  | Translational initiation factor | *infA* |
| Other genes | Subunit of Acetyl-CoA-carboxylase (fatty acid synthesis) | *accD* |
|  | c-type cytochrome synthesis gene | *ccsA (x2)* |
|  | Envelope membrane protein (carbon metabolism) | *cemA* |
|  | Protease | *clpP^b^* |
|  | Maturase (RNA processing) | *matK* |
|  | Conserved open reading frames | *ycf1 (x2), ycf2 (x2), ycf3^b^, ycf4* |

(a): Genes with one intron; (b): Genes with two introns; (x2): Duplicated genes located in IR.


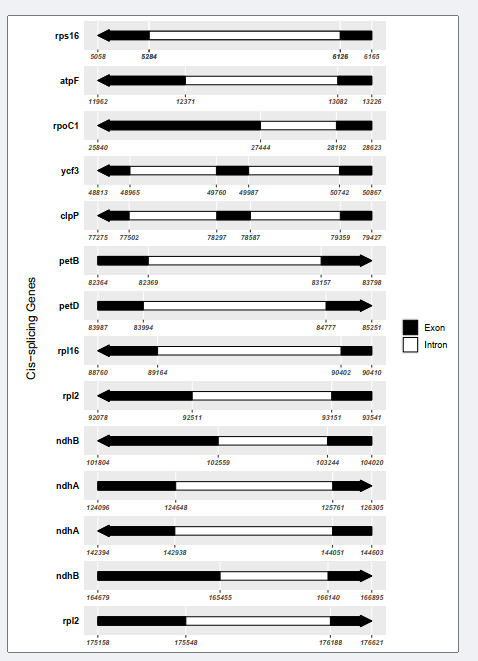


**Figure S2. Cis-splicing genes identified in the chloroplast genome of *Hoya lockii*** **V. T. Pham & Aver.** The figure shows the gene structures containing introns in the *Hoya lockii* chloroplast genome. Black boxes represent exons, and white boxes represent introns. A total of 13 protein-coding genes contain one or two introns, including *rps16, atpF, rpoC1, ycf3, clpP, petB, petD, rpl16, rpl2, ndhB,* and *ndhA*.

**
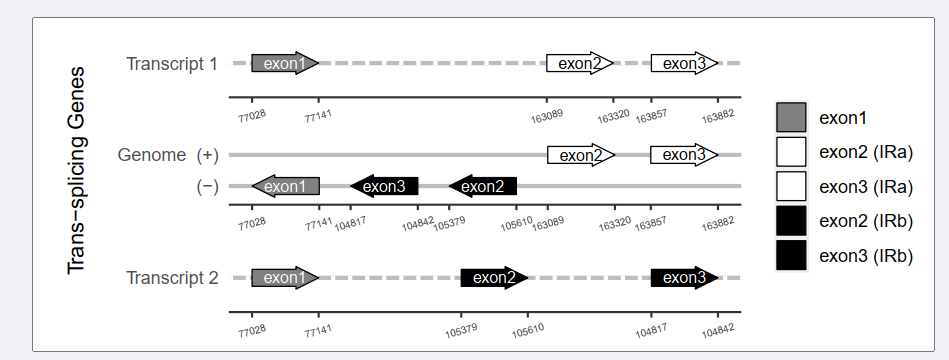
**

**Figure S3. Structure of the trans-splicing gene *rps12* in the chloroplast genome of *Hoya lockii* V. T. Pham & Aver.** The *rps12* gene is trans-spliced, with its 5′ exon located in the large single-copy (LSC) region and the duplicated 3′ exons situated within the inverted repeat regions (IRa and IRb). Exon1 is shown in grey, exons 2 and 3 in white and black corresponding to IRa and IRb, respectively. The splicing process generates two transcripts combining the separated exons into a mature mRNA.


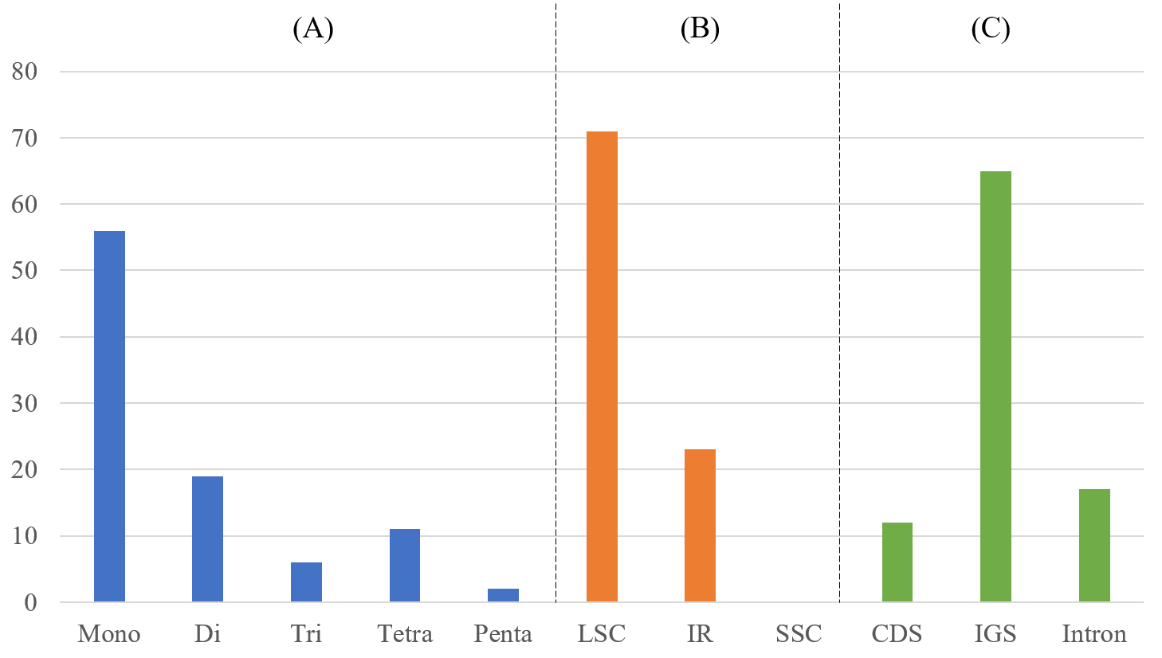


**Figure S4** Analysis of simple sequence repeats (SSRs) of *H. lockii* complete chloroplast genome. The X-axis indicates SSR types or portioned chloroplast genome regions, while the Y-axis indicates the number of SSRs. (A) Frequency of mono-, di-, tri-, tetra-, penta- nucleotide repeat types. (B) Frequency of repeat patterns in the LSC, SSC, and IR regions. **C** Frequency of repeat patterns in the CDS, IGS, and Intron regions.


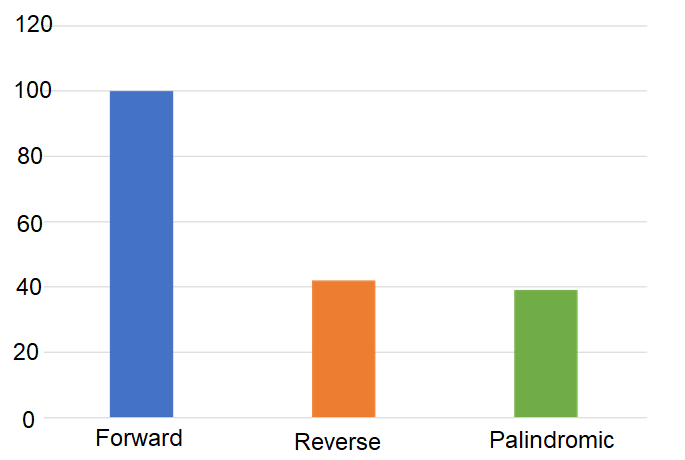


**Figure S5.** Large Sequence Repeats (LSRs) analysis on a genomic scale in *H. lockii*

**Table S3.** Relative synonymous codon usage (RSCU) for protein-coding genes in *H. lockii*

| Codon | AA | ObsFreq* | RSCU | Codon | AA | ObsFreq* | RSCU | Codon | AA | ObsFreq* | RSCU |
| --- | --- | --- | --- | --- | --- | --- | --- | --- | --- | --- | --- |
| UAG | * | 54 | 0.18 | AUC | I | 479 | 1.57 | CGC | R | 134 | 0.44 |
| UGA | * | 24 | 0.08 | AUU | I | 1,304 | 4.26 | CGG | R | 171 | 0.56 |
| UAA | * | 19 | 0.06 | AAA | K | 1,330 | 4.35 | CGU | R | 387 | 1.27 |
| GCA | A | 438 | 1.43 | AAG | K | 415 | 1.36 | AGC | S | 155 | 0.51 |
| GCC | A | 270 | 0.88 | CUA | L | 484 | 1.58 | AGU | S | 469 | 1.53 |
| GCG | A | 168 | 0.55 | CUC | L | 207 | 0.68 | UCA | S | 457 | 1.49 |
| GCU | A | 687 | 2.25 | CUG | L | 213 | 0.70 | UCC | S | 364 | 1.19 |
| UGC | C | 89 | 0.29 | CUU | L | 722 | 2.36 | UCG | S | 272 | 0.89 |
| UGU | C | 261 | 0.85 | UUA | L | 1,036 | 3.39 | UCU | S | 678 | 2.22 |
| GAC | D | 249 | 0.81 | UUG | L | 614 | 2.01 | ACA | T | 483 | 1.58 |
| GAU | D | 1,041 | 3.40 | AUG | M | 1 | 0.00 | ACC | T | 302 | 0.99 |
| GAA | E | 1,284 | 4.20 | AAC | N | 702 | 2.30 | ACG | T | 165 | 0.54 |
| GAG | E | 396 | 1.29 | AAU | N | 2 | 0.01 | ACU | T | 578 | 1.89 |
| UUC | F | 571 | 1.87 | CCA | P | 328 | 1.07 | GUA | V | 597 | 1.95 |
| UUU | F | 1,181 | 3.86 | CCC | P | 1,165 | 3.81 | GUC | V | 200 | 0.65 |
| GGA | G | 785 | 2.57 | CCG | P | 352 | 1.15 | GUG | V | 219 | 0.72 |
| GGC | G | 225 | 0.74 | CCU | P | 284 | 0.93 | GUU | V | 582 | 1.90 |
| GGG | G | 388 | 1.27 | CAA | Q | 188 | 0.61 | UGG | W | 522 | 1.71 |
| GGU | G | 637 | 2.08 | CAG | Q | 454 | 1.48 | UAC | Y | 222 | 0.73 |
| CAC | H | 181 | 0.59 | AGA | R | 804 | 2.63 | UAU | Y | 891 | 2.91 |
| CAU | H | 489 | 1.60 | AGG | R | 232 | 0.76 |  |  |  |  |
| AUA | I | 831 | 2.72 | CGA | R | 536 | 1.75 |  |  |  |  |

* ObsFreq = number of occurrences (count)


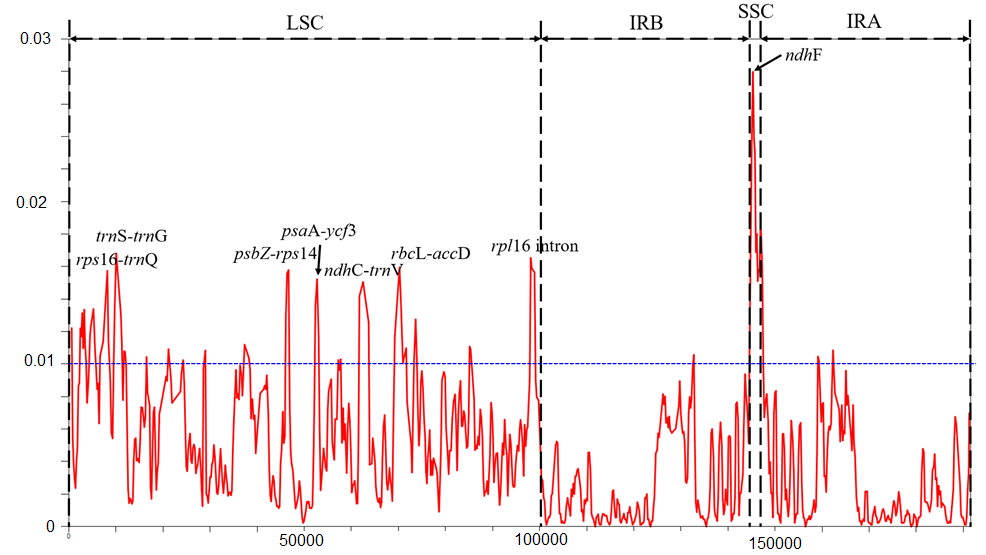


**Figure S6.** Comparative analysis of nucleotide diversity (Pi) values among the 29 *Hoya* complete cp genome sequences
